# Assessment of Health Status in Populations Living in the Semipalatinsk Nuclear Test Site Region: Results of Screening

**DOI:** 10.3390/healthcare14131988

**Published:** 2026-07-03

**Authors:** Altay Dyussupov, Galiya Alibayeva, Dariya Shabdarbayeva, Lyudmila Pivina, Nailya Chaizhunussova, Andrey Orekhov, Meruyert Massabayeva, Assel Baibussinova, Alexandra Lipikhina, Zhanargul Smailova, Gulnara Batenova, Saulesh Apbassova, Murat Lepesbayev, Saule Kozhanova, Asset Izdenov, Raushan Dosmagambetova, Tolebai Rahypbekov

**Affiliations:** 1 Office of the Rector, Semey Medical University, Semey 071400, Kazakhstan; altay.dyusupov@smu.edu.kz (A.D.); asetizdenov@mail.ru (A.I.); dosmagambetova@qmu.kz (R.D.); 2 Department of Emergency Medicine, Semey Medical University, Semey 071400, Kazakhstan; galiya.alibayeva@smu.edu.kz (G.A.); gulnara.batenova@smu.edu.kz (G.B.); 3 Office of the Vice Rector, Semey Medical University, Semey 071400, Kazakhstan; dariya.shabdarbaeva@smu.edu.kz (D.S.); zhanargul.smailova@smu.edu.kz (Z.S.); 4 Department of Public Health, Semey Medical University, Semey 071400, Kazakhstan; nailya.chaizhunusova@smu.edu.kz; 5 Department of Internal Medicine, Semey Medical University, Semey 071400, Kazakhstan; 6 Research Laboratory Center, Semey Medical University, Semey 071400, Kazakhstan; meruyert.massabayeva@smu.edu.kz; 7 Department of Biostatistics, Semey Medical University, Semey 071400, Kazakhstan; assel.baibussinova@smu.edu.kz; 8 Research Institute of Radiation Medicine and Ecology, Semey Medical University, Semey 071400, Kazakhstan; a.v.lipikhina@mail.ru; 9 Department of Pathology, Semey Medical University, Semey 071400, Kazakhstan; saulesh.apbasova@smu.edu.kz (S.A.); king87ml@gmail.com (M.L.); 10 Department of Anatomy, Semey Medical University, Semey 071400, Kazakhstan; saule.kozhanova@smu.edu.kz; 11 Office of the Rector, West Kazakhstan Marat Ospanov State Medical University, Aktobe 030019, Kazakhstan; tolebai.rahypbekov@mail.ru

**Keywords:** Semipalatinsk Nuclear Test Site, ionizing radiation, radiation exposure, population screening, arterial hypertension, thyroid disorders, metabolic disorders, dose–response relationship

## Abstract

**Background**: The Semipalatinsk Nuclear Test Site (SNTS) represents a unique example of long-term population exposure to ionizing radiation. This study aimed to assess the health status of individuals from three generations permanently residing in territories affected by radioactive contamination resulting from nuclear weapons testing at the SNTS, based on the findings of a population screening examination. **Materials and Methods:** A cross-sectional screening study was conducted among 2802 adults aged 18 years and older. The exposed group consisted of residents of the Abay and Beskaragai districts of the Abay Region and their descendants (*n* = 1358). The control group included residents of Arshaly village in the Akmola Region with no history of radiation exposure (*n* = 1444). All participants underwent a structured questionnaire survey, clinical examination, biochemical and hematological testing, and assessment of thyroid function. Individual radiation doses were obtained from the State Scientific Automated Medical Registry of Persons Exposed to Radiation (SSAMR). **Results:** Arterial hypertension (*p* < 0.001), chronic ischemic heart disease (*p* < 0.001), thyroid disorders (*p* < 0.001), malignant neoplasms (*p* = 0.003), renal diseases, and respiratory diseases were significantly more prevalent in the exposed population than in the control group. Exposed individuals also demonstrated significantly higher levels of total cholesterol, triglycerides, and the triglyceride-glucose (TyG) index, indicating increased insulin resistance. After adjustments, the only outcome that retained a statistically significant association with radiation dose was thyroid disorders (adjusted OR per 10 mSv increase = 1.017; 95% CI: 1.009–1.025; *p* < 0.001). ROC analysis demonstrated a moderate discriminative ability of radiation dose with respect to arterial hypertension (AUC = 0.715), chronic ischemic heart disease (AUC = 0.735), and ischemic stroke (AUC = 0.711). **Conclusions:** The findings suggest long-term adverse health effects associated with radiation exposure among populations residing near the SNTS. Continued epidemiological surveillance and medical monitoring of exposed individuals and their descendants are warranted.

## 1. Introduction

The Semipalatinsk Nuclear Test Site (SNTS) represents a unique example of long-term population exposure to ionizing radiation and remains one of the largest environmental radiation incidents in human history. Nuclear weapons testing was conducted at the site for four decades, resulting in extensive radioactive contamination across large areas of northeastern Kazakhstan. Consequently, more than one million people residing in affected territories were exposed to both external and internal sources of ionizing radiation [1,2,3,4,5].

The most intensive radiation exposure occurred during the period of atmospheric and surface nuclear testing between 1949 and 1965, when residents experienced repeated episodes of acute external irradiation combined with prolonged chronic internal exposure across a wide range of doses. Although underground nuclear testing was introduced later, radiation exposure did not cease entirely. Until the late 1980s, some underground tests were accompanied by the release of radioactive gases into the environment, resulting in additional exposure of nearby populations, including children [6,7].

Comprehensive investigation of the health consequences of radiation exposure among populations living near the SNTS became feasible only in the early 1990s, following the gradual availability of information regarding nuclear test characteristics, environmental contamination patterns, and reconstructed individual and population radiation doses. Historical evidence indicates that, as early as the 1960s, Dispensary No. 4 of the USSR Ministry of Health had documented health effects associated with several atmospheric nuclear explosions conducted in 1949, 1951, 1953, and 1956. Residents of settlements located in the Abay, Abraly, Beskaragai, and Zhanasemey districts of the former Semipalatinsk region were affected by radioactive fallout. The institution also possessed data on radiation dose assessments conducted by specialists from the test site and the Institute of Biophysics of the USSR Ministry of Health [8,9].

Most studies evaluating the health effects of radiation exposure in various radioecological settings have focused on the consequences of relatively high radiation doses [2,10,11,12]. In contrast, the health effects for the population of different age groups associated with low- and moderate-dose exposure, remain insufficiently understood. Existing knowledge has largely been derived from studies of survivors of the atomic bombings of Hiroshima and Nagasaki, populations affected by the Chernobyl nuclear accident, and individuals exposed to medical sources of ionizing radiation [13,14,15,16,17,18,19,20]. However, findings from these studies remain inconsistent and, in some cases, contradictory.

Previous investigations of populations residing near the SNTS have primarily focused on cancer incidence [21,22], cardiovascular diseases [23], and mortality outcomes. Nevertheless, comprehensive assessments of overall health status among residents of radiation-affected territories remain limited, with most studies addressing only specific diseases or selected preclinical conditions [24,25]. In 2018, the results of first screening study on circulatory diseases in a population exposed to radiation were published [26].

Health screening is considered one of the most effective approaches for evaluating the long-term health consequences of radiation exposure in affected populations. Screening programs facilitate the early detection of both malignant and non-malignant conditions potentially associated with ionizing radiation, including thyroid disorders, cardiovascular diseases, and hematological abnormalities [27,28,29,30]. In addition to identifying previously undiagnosed conditions, screening results provide valuable data for the development and maintenance of specialized health registries, enabling long-term epidemiological surveillance, personalized follow-up programs, and targeted preventive interventions [31,32,33].

The design and effectiveness of screening programs depend on several factors, including radiation dose, age at exposure, sex, and time elapsed since exposure. Individuals exposed during childhood, particularly during prenatal development, are generally considered to be among the most vulnerable population groups [34,35]. In the context of the SNTS, many residents experienced both external and internal radiation exposure during critical periods of growth and development throughout the years of nuclear weapons testing [2]. Furthermore, increasing attention has been directed toward the potential long-term health consequences among the descendants of exposed individuals, who currently constitute a substantial proportion of the region’s economically active and reproductive-age population.

Therefore, the aim of our study was to assess the health status of three generations of individuals permanently residing in territories affected by nuclear weapons testing at the Semipalatinsk Nuclear Test Site, based on the results of population screening examination. Additionally, we aimed to evaluate the relationship between individual radiation exposure dose and key health indicators identified during the screening.

## 2. Materials and Methods

### 2.1. Participant Recruitment and Screening Procedures

The primary source for participant identification and recruitment was the State Scientific Automated Medical Registry (SSAMR) of Persons Exposed to Radiation. The SSAMR was established in 2002 through collaboration between Kazakhstan and the Radiation Effects Research Foundation (RERF, Japan), with support from the Ministry of Health of the Republic of Kazakhstan [36].

At present, the SSAMR contains records for 374,249 individuals, including directly exposed persons and their descendants, regardless of current vital status (living, deceased, or migrated). The registry includes demographic information, residential and exposure histories, vital status, educational attainment, occupation, official identification records, and medical data obtained through comprehensive health examinations. For deceased individuals, information on the underlying cause of death is also available. Each registry member is assigned a unique identification number, enabling linkage of all available demographic, clinical, and exposure-related information.

The study involved residents of the Abay and Beskaragay districts of the Abay region of the Republic of Kazakhstan, who were exposed to radiation as a result of nuclear weapons testing at the Semipalatinsk Nuclear Test Site (SNT), as well as their descendants in two subsequent generations. A list of 1500 prospective study participants was selected from the database of the State Scientific Automated Medical Registry of Persons Exposed to Radiation. The study group included residents of six settlements located near the test site and exposed to radiation in approximately equal doses: Dolon, Mostik, and Cheremushki in the Beskaragay district, and Karaul, Sarzhal, and Kainar in the Abay district. The inclusion of six villages was motivated by the fact that, due to natural population decline and migration, each of them currently has a small number of permanently residing families associated with nuclear weapons testing.

An equal number of residents of the village of Arshaly in the Akmola region of the Republic of Kazakhstan, with no history of exposure to radiation from major nuclear or radiological incidents, were selected as a control group. Residents of this village were selected as the comparison group because it shares broadly similar climatic, socioeconomic, and healthcare characteristics with the exposed settlements, according to official data from the Bureau of National Statistics of the Republic of Kazakhstan [https://stat.gov.kz/en/, accessed on 30 May 2025].

Exclusion criteria included organic diseases of the central nervous system, decompensated chronic somatic diseases, and a history of hepatitis B or hepatitis C. Each participant completed a standardized questionnaire, including written informed consent and a structured questionnaire covering their medical history, chronic diseases, sociodemographic characteristics, lifestyle factors, and health-related behaviors.

The present screening study was aimed at assessing the health status of individuals of different age groups with different ranges of radiation doses as a whole, rather than assessing individual classes of diseases, as, for example, was previously carried out in relation to cardiovascular diseases. Screening examinations were conducted by a multidisciplinary team of physicians from medical institutions in Semey, including cardiologists, neurologists, endocrinologists, and internists. Trained nurses assisted in data collection and collected blood and urine samples for laboratory testing. Laboratory tests included a complete blood count, biochemistry panel, thyroid hormone levels, and routine urine analysis. Diagnoses were established based on anamnesis, physical examination data, laboratory tests and visual diagnostic methods.

Diagnoses were established by qualified physicians in accordance with current national and international clinical guidelines based on a comprehensive assessment that included medical history, physical examination, laboratory investigations, and instrumental examinations. Cardiovascular diseases were diagnosed based on clinical history, repeated blood pressure measurements, electrocardiography, and, where indicated, echocardiography. Thyroid disorders were identified using thyroid ultrasonography together with laboratory assessment of thyroid-stimulating hormone (TSH), free triiodothyronine (FT3), free thyroxine (FT4), and anti-thyroid peroxidase antibodies (anti-TPO). Diabetes mellitus was diagnosed on the basis of fasting plasma glucose levels and/or a previously established diagnosis or the use of glucose-lowering therapy. Chronic kidney disease was assessed using serum creatinine, estimated glomerular filtration rate (eGFR), urinalysis findings, and, where indicated, renal ultrasonography. Respiratory and gastrointestinal diseases were diagnosed based on clinical evaluation and relevant laboratory or instrumental investigations when clinically indicated. Diagnoses of malignant neoplasms were confirmed using available medical records or the results of previous histopathological examinations, where available.

**Figure 1:**
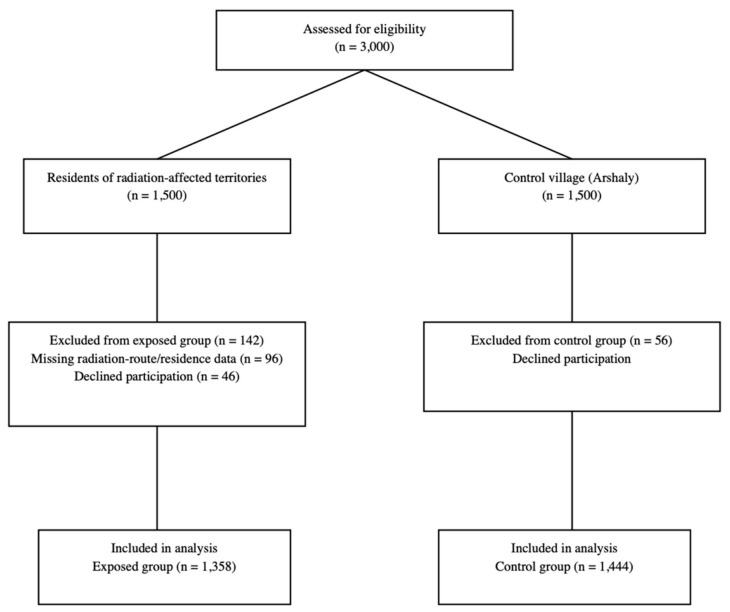
Flow diagram of participant recruitment and inclusion in the study.

**Figure 2:**
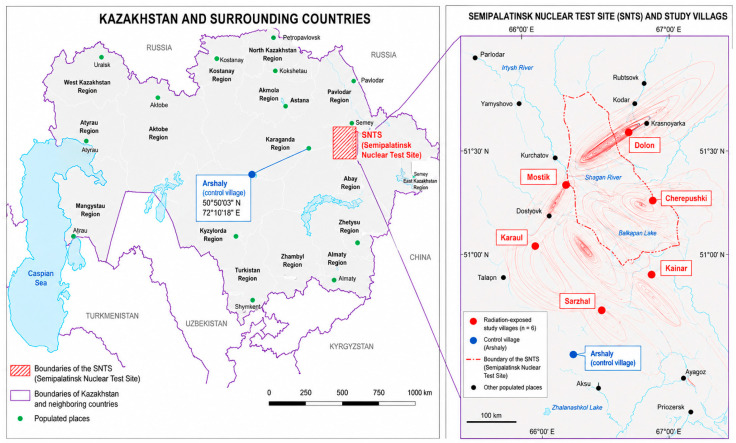
Study areas and participant recruitment sites.

From the 3000 pre-screened residents, a representative sample of 2802 adults aged 18–75 years was recruited. After excluding participants with incomplete data and those who declined to participate for various reasons, the final analytical sample included 1358 exposed individuals and 1444 controls (Figure 1 and Figure 2).

The sample size was determined based on the population size of the study districts and expected disease prevalence estimates reported in previous studies [37,38,39]. Sample size calculations were performed using OpenEpi software (Version 3.01).

All patients confirmed their consent to participate in the study and the results to be published. The study was approved by the Ethics Committee of Semey Medical University (protocol №2b, 18 December 2024).

### 2.2. Radiation Dose Assessment

Assessment of the health effects of ionizing radiation requires reliable estimation of individual radiation doses. Effective dose calculations provide an objective basis for evaluating radiation-related health risks and characterizing exposure levels within large population groups.

Individual radiation doses were obtained using the automated dose reconstruction system implemented within the State Scientific Automated Medical Registry (SSAMR) of Persons Exposed to Radiation. According to the Methodological Recommendations of the Republic of Kazakhstan [40], the fundamental criterion for individual dose assessment is the determination of the contribution of both external gamma radiation exposure and internal radiation exposure resulting from the intake of long-lived radionuclides into the human body through in-halation of contaminated air during the passage of radioactive clouds, as well as through the consumption of contaminated drinking water and locally produced food products. Based on contemporary scientific evidence regarding the relative contributions of acute and chronic radiation exposure under conditions similar to those surrounding the Semipalatinsk Nuclear Test Site (SNTS), the following approach has been proposed for estimating cumulative radiation dose: external exposure is assumed to contribute 80% of the maximum possible dose, whereas internal exposure is assumed to contribute 20% of the maximum possible dose.

The maximum possible dose is defined as the highest population exposure dose established by the Law of the Republic of Kazakhstan [41] according to residence in a specific settlement affected by the activities of the SNTS. Calculation of individual radiation doses takes into account each participant’s date of birth, age-specific behavioral factors, occupation, and residential history, including the end date of residence in each settlement, corresponding either to migration from the settlement or to death.

Dose reconstruction included all settlements in which study participants resided during the period 1949–1990 and which were affected by radioactive fallout resulting from nuclear weapons testing at the SNTS. Periods of residence outside the affected territories, including residence in remote regions of Kazakhstan or abroad, were not included in dose estimation because these locations were not affected by radioactive fallout from nuclear tests conducted at the SNTS. The median radiation dose among all exposed participants was 138.0 mSv (IQR: 45.2–238.0 mSv). Individuals born before 1962, during the period of atmospheric and surface nuclear testing, had the highest cumulative doses, with a median of 454.0 mSv (IQR: 257.0–789.0 mSv). Participants born between 1963 and 1989 had substantially lower doses, with a median of 118.0 mSv (IQR: 56.0–158.0 mSv). Individuals born after 1990 were considered unexposed to direct radiation from nuclear testing activities (Table 1). The highest radiation doses (up to 1000 mSv) were observed in the group of individuals born before 1962, but those born between 1963 and 1989 also had a maximum dose of 676.0 mSv. Although individuals born after the cessation of nuclear testing had a median dose of zero, some study participants in this group had a dose of over 7 mSv. The median age in the first group was 69 (IQR: 65–74) years, in the second group 53 (IQR: 46–58) years, and in the third 29 (IQR: 24–33) years. Given the cross-sectional nature of the study, it was not possible for us to trace the transgenerational effects of radiation across generations.

### 2.3. Laboratory Methods

All laboratory analyses were performed at the Research Laboratory Center of Semey Medical University.

Hematological Analysis. Complete blood counts (CBCs) were performed to evaluate peripheral blood cell composition and identify potential hematological abnormalities. Venous blood samples were collected in the morning after an overnight fast into EDTA-containing vacuum tubes (EcoPharm LLP, Almaty, Kazakhstan). Hematological parameters were measured using a D7-CRP automated hematology analyzer (Shenzhen Dymind Biotechnology Co., Ltd., Shenzhen, China). All analyses were conducted according to the manufacturer’s protocols using certified reagents, calibration procedures, and quality-control materials.

Biochemical Analysis. Biochemical testing was performed to assess metabolic status and organ function, including markers of liver function, kidney function, carbohydrate metabolism, and lipid metabolism. Fasting venous blood samples were collected into serum-separating tubes containing a clot activator. Following clot formation, samples were centrifuged at 3000 rpm for 10 min, and serum was analyzed using an EXC 200 automated biochemical analyzer (Shanghai Medical Technology Co., Ltd., Shanghai, China).

The following biochemical parameters were measured: glucose, urea, creatinine, total and direct bilirubin, alkaline phosphatase, alanine aminotransferase, aspartate aminotransferase, gamma-glutamyl transferase, total protein, albumin, uric acid, total cholesterol, and triglycerides. Reference values recommended by the manufacturer were used for interpretation of laboratory findings.

Thyroid function and autoimmune thyroid status were evaluated by measuring serum concentrations of thyroid-stimulating hormone (TSH), free thyroxine (FT4), free triiodothyronine (FT3), and thyroid peroxidase antibodies (anti-TPO).

Fasting venous blood samples were collected and processed according to standard laboratory procedures. Hormone concentrations were measured using a T1000 automated chemiluminescence analyzer (Takurra, Ust-Kamenogorsk/Oskemen, Kazakhstan). Certified reagent kits supplied by Teco Diagnostics (Anaheim, CA, USA) and Mindray Bio-Medical Electronics Co., Ltd. (Shenzhen, China) were used. Internal quality-control procedures and routine calibration were performed daily.

Reference intervals were as follows: TSH 0.4–4.0 mIU/L, FT4 9.0–22.0 pmol/L, FT3 2.6–5.7 pmol/L, and anti-TPO <35 IU/mL (Table 2).

### 2.4. Statistical Analysis

Statistical analyses were performed using SPSS Statistics version 20.0 (IBM Corp., Armonk, NY, USA), Jamovi version 2.5, and R version 4.5.1 (R Foundation for Statistical Computing, Vienna, Austria). The distribution of continuous variables was assessed using the Shapiro–Wilk test. Because most variables were not normally distributed, continuous data are presented as median and interquartile range (IQR). Comparisons between two independent groups were performed using either the Mann–Whitney U test or Student’s *t*-test, as appropriate. Comparisons involving three or more groups were conducted using the Kruskal–Wallis test with Bonferroni-adjusted post hoc comparisons or one-way analysis of variance (ANOVA) followed by appropriate post hoc testing. Categorical variables are presented as frequencies and percentages and were compared using Pearson’s chi-square (χ^2^) test.

Associations between radiation dose and health outcomes were evaluated using linear and logistic regression models. Regression coefficients, odds ratios (ORs), and corresponding 95% confidence intervals (95% CIs) were calculated. Spearman’s rank correlation analysis was performed to assess relationships between radiation exposure levels and clinical or laboratory parameters. Receiver operating characteristic (ROC) curve analysis was used to evaluate the discriminatory performance of radiation dose for predicting selected health outcomes. The area under the ROC curve (AUC), sensitivity, and specificity were calculated.

A two-sided *p*-value < 0.05 was considered statistically significant.

## 3. Results

The sociodemographic characteristics of the study participants are presented in Table 3. The median age was comparable between the exposed and control groups, at 54.0 and 53.0 years, respectively, with no statistically significant difference observed. Women predominated in both groups and accounted for the majority of participants in the control group (82.3%, *p* < 0.001). In both study groups, more than one-third of participants had completed secondary education; however, the proportion of individuals with higher education was significantly greater in the control group (*p* < 0.001). The majority of participants in both groups were employed in occupations not associated with hazardous working conditions and were married. More than 80% of participants reported never having smoked, and approximately half reported no alcohol consumption. However, 42% of participants in the exposed group declined to provide information regarding alcohol use, which limits the interpretation of these findings.

The prevalence of several chronic diseases was significantly higher in the exposed group than in the control group. Specifically, arterial hypertension, overall circulatory system diseases, chronic ischemic heart disease, valvular heart disease, ischemic stroke, chronic cerebrovascular disease, thyroid disorders, malignant neoplasms, and gastrointestinal diseases were observed significantly more frequently among individuals residing in radiation-affected areas. In contrast, diabetes mellitus was significantly more prevalent in the control group (Table 4). The comparisons presented in Table 4 represent unadjusted descriptive analyses based on Pearson’s χ^2^ test and do not account for potential confounding factors. Adjusted associations were assessed separately using multivariable regression models.

Analysis of biochemical parameters revealed that the median values of most indicators remained within the corresponding reference ranges in both study groups (Table 5). Nevertheless, several statistically significant differences were observed between the groups. Participants in the exposed group exhibited significantly higher median levels of total bilirubin, total cholesterol, and triglycerides compared with controls. In contrast, serum concentrations of uric acid, creatinine, urea, alkaline phosphatase, aspartate aminotransferase (AST), gamma-glutamyl transferase (GGT), glucose, and alanine aminotransferase (ALT) were significantly higher in the control group. Assessment of insulin resistance using the triglyceride-glucose (TyG) index demonstrated significantly higher values among radiation-exposed individuals, suggesting a less favorable metabolic profile in this population.

Of particular interest to us was the analysis of the thyroid gland status of the study subjects, as this organ is well known to be highly sensitive to radiation exposure. In the main study group, statistically significantly elevated median values for thyroid peroxidase antibodies and thyroid-stimulating hormone, as well as decreased thyroxine levels, were found, indicating decreased thyroid function due to a possible autoimmune process (Table 6).

Table 7 summarizes the biochemical and thyroid-related laboratory parameters according to birth cohort, which also reflects different levels of radiation exposure risk. Participants born before 1963, during the period of atmospheric and surface nuclear testing, experienced the highest levels of direct external radiation exposure as well as prolonged internal exposure. Individuals born between 1963 and 1989 were primarily exposed to lower cumulative doses associated with the period of underground nuclear testing, whereas participants born after 1990 had no direct exposure to radiation from nuclear testing and represented the third generation of descendants of exposed individuals. Analysis of laboratory parameters revealed significant differences between birth cohorts only for thyroid hormone levels. The lowest concentrations of free thyroxine (FT4) and free triiodothyronine (FT3), approaching values consistent with a hypothyroid state, were observed among participants born before 1963 who had experienced direct radiation exposure. Among individuals born between 1963 and 1989, thyroid hormone levels remained within the reference range but were closer to its lower limit. Notably, serum thyroid peroxidase antibody (anti-TPO) levels among descendants of exposed individuals were approximately twofold higher than those observed in the oldest directly exposed cohort. This finding may indicate persistent alterations in thyroid autoimmunity among subsequent generations and warrants further investigation.

Among the laboratory parameters studied, statistically significant differences between age groups were found only for T3 and T4 levels (in both cases, *p* < 0.001), as well as glucose. Pairwise comparisons with the Bonferroni correction revealed that participants born before 1963 had statistically significantly lower FT3 and FT4 levels compared to the two younger groups. At the same time, there were no differences between the groups born between 1963 and 1989 and after 1990. Despite the identified differences, median hormone values remained within the reference range in most cases. Among participants in the exposed group born before 1962, FT3 levels below the laboratory reference range (<2.6 pmol/L) were observed in 165 of 354 participants (46.6%), whereas FT4 levels below the reference range (<9.0 pmol/L) were identified in 104 of 358 participants (29.1%). To assess the potential influence of extreme observations, a sensitivity analysis was performed after excluding statistical outliers defined according to Tukey’s criterion (values lying beyond 1.5 × the interquartile range). Exclusion of these outliers did not result in any substantial changes in the distribution of thyroid hormone levels.

For the remaining parameters, including creatinine, total cholesterol, triglycerides, TyG index, thyroid-stimulating hormone (TSH), and thyroid peroxidase antibodies (anti-TPO), no statistically significant differences were found between the study groups after accounting for multiple comparisons. Although anti-TPO levels in the offspring of the irradiated population were somewhat higher compared to the directly irradiated group, these differences did not reach statistical significance.

An assessment of the relationship between the radiation dose and diagnosed diseases, carried out using regression analysis, demonstrated its statistically significant relationship with hypertension (OR = 1.104), chronic ischemic heart disease (OR = 1.026), ischemic and hemorrhagic strokes (OR = 1.026; OR = 1.022, respectively), malignant neoplasms (OR = 1.025), respiratory diseases (OR = 1.017), renal diseases (OR = 1.012), and thyroid disorders (OR = 1.007) (Table 8).

After adjustments, most of the associations between radiation dose and disease outcomes previously identified in the univariate analysis were significantly weakened and lost statistical significance. The only outcome that retained a statistically significant association with radiation dose was thyroid disorders (adjusted OR = 1.017; 95% CI: 1.009–1.025; *p* < 0.001). There was also a trend toward maintaining statistical significance after adjustments for chronic ischemic heart disease (*p* = 0.071). Education level and marital status were not available for all participants in a format suitable for inclusion in regression models. Data on alcohol consumption contained a significant proportion of missing values, particularly in the exposure group, and therefore this variable was not included in the adjusted models.

**Figure 3:**
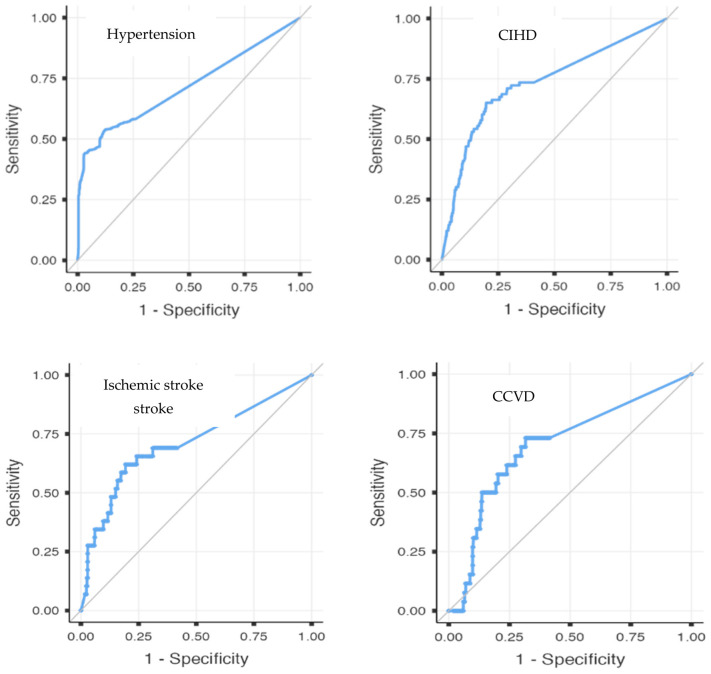
ROC curve in the model of disease development in exposed people (AUC > 0.7).

Analysis of the ROC curve and AUC in the model of disease development depending on the radiation dose showed moderate predictive ability of the model with respect to such diseases as hypertension (the area under the ROC curve for AUC was 0.715 (95% CI 0.698; 0.732, *p* < 0.001)); chronic ischemic heart disease (the area under the ROC curve for AUC was 0.735 (95% CI 0.675; 0.795, *p* < 0.001)); ischemic strokes (the area under the ROC curve for AUC was 0.711 (95% CI 0.640; 0.819, *p* < 0.001)) and chronic cerebrovascular diseases (the area under the ROC curve for AUC was 0.700 (95% CI 0.599; 0.801, *p* < 0.001)). The area under the ROC curve for AUC was significantly lower, but statistically significant for respiratory diseases (0.646; 95% CI 0.592; 0.700), thyroid disorders (0.540; 95% CI 0.513; 0.576), gastrointestinal diseases (0.596; 95% CI 0.563; 0.628), renal diseases (0.574; 95% CI 0.540; 0.607) and malignant neoplasms (0.673; CI 0.586; 0.760) (Figure 1 and Figure 3, Table 9).

As a supplementary analysis, radiation dose was categorized into predefined exposure groups (0, <500 mSv, and 500–1000 mSv) to assess potential non-linear or threshold dose–response associations. Logistic regression analysis adjusted to age, sex and smoking status showed that radiation exposure in the dose range both up to 500 mSv and greater than 500 mSv had statistically significant associations with some diseases (Table 10). Thus, in the group with a dose <500 mSv, the likelihood of developing hypertension was more than 3 times higher (OR = 3.61; 95% CI: 2.96–4.39; *p* < 0.001), and chronic ischemic heart disease was almost 3 times higher (OR = 2.66; 95% CI: 1.548–4.593; *p* < 0.001). Similarly, for chronic cerebrovascular diseases, a more than 4-fold increase in risk was noted (OR = 6.26; 95% CI: 2.35–16.63; *p*< 0.001). For thyroid disorders, the OR was 1.421 (1.138–1.775, *p* = 0.002), for gastrointestinal diseases—1.71 (1.32–2.2, *p* < 0.01), renal diseases—1.41 (1.08–1.83, *p* = 0.009) and respiratory diseases—2.2 (1.42–3.45, *p* < 0.01). For the dose range of 500–1000 mSv, the adjusted ORs also showed statistical significance for hypertension—9.88 (3.95–24.68, *p* < 0.01), chronic ischemic heart disease—3.44 (1.737–6.81, *p* < 0.01), ischemic stroke—6.15 (1.99–18.91, *p* = 0.02), thyroid disorders—2.747 (1.798–4.196, *p* < 0.01), malignant neoplasms—3.52 (1.45–8.54, *p* = 0.005), gastrointestinal diseases—1.65 (1.03–2.64, *p* = 0.037), renal diseases—2.09 (1.31–3.32, *p* = 0.002) and respiratory diseases—2.46 (1.22–4.98, *p* = 0.012).

## 4. Discussion

The findings of the present study indicate that significantly higher prevalence of arterial hypertension, chronic ischemic heart disease, valvular heart disease, ischemic stroke, chronic cerebrovascular disease, thyroid disorders, malignant neoplasms, and gastrointestinal diseases was observed among individuals residing in radiation-affected areas.

Participants in the exposed group exhibited significantly higher median levels of total bilirubin, total cholesterol, triglycerides, and the triglyceride–glucose (TyG) index, a surrogate marker of insulin resistance, compared with controls. Taken together, these findings suggest the presence of long-term metabolic alterations in the exposed population.

The findings related to thyroid function were less consistent and should therefore be interpreted with caution. Laboratory analyses demonstrated lower concentrations of free thyroxine (FT4) and free triiodothyronine (FT3), particularly among participants born before the cessation of atmospheric and surface nuclear testing, who experienced the highest levels of direct radiation exposure. These findings highlight the need for further research to clarify the long-term and potential effects of radiation exposure on thyroid health.

One of the most important findings of the present study was the identification of significant associations between radiation dose and several major chronic diseases. Multivariable regression analyses demonstrated significant dose-dependent relationships with arterial hypertension, chronic ischemic heart disease, ischemic and hemorrhagic stroke, thyroid disorders, malignant neoplasms, respiratory diseases, and renal diseases. However, after adjusting for gender, age, and smoking status, a statistically significant association was observed only for thyroid disorders; for chronic ischemic heart disease, the association was only at the trend level. For the remaining diseases, a weakening association with radiation dose was observed, with a loss of statistical significance. Notably, these associations were observed at cumulative radiation doses below 500 mSv, suggesting that even relatively moderate levels of long-term radiation exposure may contribute to an increased risk of chronic disease development.

Receiver operating characteristic (ROC) analysis demonstrated that radiation dose had the highest discriminatory performance for arterial hypertension, chronic ischemic heart disease, ischemic stroke, and chronic cerebrovascular disease. Although the area under the ROC curve (AUC) was lower for respiratory diseases, thyroid disorders, gastrointestinal diseases, renal diseases, and malignant neoplasms, the observed associations remained statistically significant.

The present study contributes to a better understanding of the long-term health consequences of radiation exposure and provides additional evidence regarding the biological mechanisms that may underlie chronic disease development in radiation-exposed populations. Over recent decades, research on the health effects of nuclear testing at the Semipalatinsk Nuclear Test Site (SNTS) has evolved substantially, progressing from descriptive clinical observations and radiation dose reconstruction studies to large-scale epidemiological, cohort-based, and molecular genetic investigations [42]. More recently, increasing attention has been directed toward the assessment of potential effects of radiation exposure and the risk of developing chronic non-communicable diseases among the descendants of exposed individuals.

Our findings are consistent with those of a previous screening study conducted in 2018 among approximately 2000 residents of radiation-affected territories surrounding the SNTS. That study reported a higher prevalence of arterial hypertension among radiation risk groups and demonstrated a dose-dependent increase in age-adjusted hypertension risk [43].

Comparison of our results with findings from the Japanese Life Span Study (LSS) and related cohorts of atomic bomb survivors and their descendants is of particular interest [44,45]. An important consideration in this comparison is the substantial difference in radiation exposure levels between the studied populations. According to published data, 45.0% of participants in the Japanese cohort received estimated doses of ≤5 mGy, while 34.2% received doses ranging from 5 to 99 mGy [46]. These exposure levels were considerably lower than those observed among many participants in our study, particularly among individuals born before the 1990s.

Although direct comparisons between populations should be interpreted cautiously because of differences in exposure patterns, dosimetry methods, and study design, these findings support the possibility that relatively moderate levels of long-term radiation exposure may be associated with adverse health outcomes. This issue is particularly relevant for populations residing near the SNTS, where radiation exposure was chronic, heterogeneous, and occurred through both external and internal pathways.

Previous analyses of Japanese atomic bomb survivor cohorts have demonstrated statistically significant associations between radiation exposure and several non-cancer outcomes, including circulatory and respiratory diseases. It should be noted, however, that in the Life Span Study (LSS), risk estimates for non-cancer diseases were derived primarily from mortality data, which introduces certain methodological limitations. Despite these limitations, the investigators reported increased excess relative risks (ERRs) for cardiovascular diseases and stroke overall. In contrast, statistically significant associations were not consistently observed for specific disease entities, including coronary heart disease and individual stroke subtypes [47,48,49,50].

The findings of the Adult Health Study (AHS), a clinical subcohort of 24,358 participants from the LSS, are particularly relevant in the context of the present investigation [51,52]. Unlike the LSS mortality analyses, the AHS focused on morbidity outcomes identified through periodic clinical examinations and included detailed assessments of cardiovascular risk factors and biochemical parameters [52,53,54]. Results from the AHS demonstrated radiation-associated changes not only in cancer incidence but also in the cardiovascular system, endocrine organs, liver, and ocular tissues. Moreover, subsequent analyses reported an increased risk of hemorrhagic stroke, including subarachnoid hemorrhage, among exposed individuals [54].

Of particular interest are the findings regarding serum cholesterol levels among Japanese atomic bomb survivors [55]. Dyslipidemia is widely recognized as a major contributor to cardiovascular disease development, and previous studies have reported modest but statistically significant increases in total cholesterol levels among irradiated individuals. At a dose of 1 Gy, total cholesterol concentrations were approximately 2.3–2.5 mg/dL higher in women and 1.4–1.6 mg/dL higher in men compared with non-exposed individuals. These observations led the authors to suggest that ionizing radiation may contribute to long-term metabolic alterations that persist for decades after exposure.

Our findings are broadly consistent with these observations. Taken together, these findings support the hypothesis that long-term radiation exposure may be associated with persistent metabolic alterations. Such changes could potentially contribute to an increased burden of cardiovascular and metabolic diseases through mechanisms involving lipid dysregulation, endothelial dysfunction, chronic inflammation, and hormonal imbalance. However, the biological pathways underlying these associations require further investigation.

Findings related to overt and subclinical thyroid diseases are of particular interest in the context of radiation epidemiology. Previous studies by Imaizumi and colleagues demonstrated that radiation exposure was primarily associated with the development of thyroid nodules, whereas autoimmune thyroiditis, hypothyroidism, and Graves’ disease did not show consistent dose-dependent relationships [56,57,58]. In contrast, significant linear dose–response associations were observed for thyroid malignancies as well as benign thyroid nodules and cysts (*p* < 0.001). The authors estimated that approximately 28% of solid thyroid nodules, 37% of thyroid cancers, 31% of benign nodules, and 25% of thyroid cysts could be attributed to radiation exposure at a mean thyroid dose of 0.449 Sv [56].

Studies conducted among radiation-exposed populations in Kazakhstan have likewise reported significant dose–response relationships for thyroid diseases, including both malignant and autoimmune thyroid disorders (OR = 1.42; 95% CI: 1.22–1.65; *p* < 0.001) [59]. In the previous study, although differences in thyroid hormone concentrations and anti-thyroid peroxidase antibody (anti-TPO) levels were observed among specific birth groups, multivariable analyses did not demonstrate a consistent direct association between radiation dose and thyroid dysfunction. These findings suggest that factors other than radiation exposure may contribute to the observed variations in thyroid status and underscore the need for further investigations into the long-term and potential intergenerational effects of ionizing radiation on thyroid health.

The results of the Korean Atomic Bomb Survivor Cohort (K-ABC) study also provide valuable insights into the long-term and transgenerational consequences of radiation exposure [60]. This cohort included 2544 individuals, comprising 1109 atomic bomb survivors (first generation, G1), 1193 children of survivors (second generation, G2), and 242 grandchildren of survivors (third generation, G3). The study demonstrated that directly exposed survivors exhibited the highest prevalence of chronic non-communicable diseases. Arterial hypertension was reported in 55.7% of G1 participants, compared with 24.8% and 1.7% in the second and third generations, respectively (*p* < 0.001). Similarly, diabetes mellitus was observed in 29.6% of G1 individuals, compared with 13.5% in G2 and 1.7% in G3 (*p* < 0.001). Cancer prevalence decreased across generations, from 16.7% in G1 to 9.2% in G2 and 1.2% in G3 (*p* < 0.001). Thyroid disorders were also more common among directly exposed survivors, affecting 14.4% of G1 participants, compared with 11.8% and 3.3% of individuals in the second and third generations, respectively (*p* < 0.001). The second generation additionally demonstrated a higher prevalence of certain reproductive disorders.

Although direct comparisons between the Korean and Kazakhstani populations should be interpreted cautiously because of differences in exposure conditions and study design, our findings similarly indicate a substantial burden of chronic non-communicable diseases among individuals born during periods of active nuclear testing and among those born before 1990, who experienced varying degrees of radiation exposure during the era of underground testing. Together, these observations highlight the importance of continued monitoring of both directly exposed individuals and their descendants.

Additional insights into the potential biological mechanisms underlying radiation-associated disease development have been provided by the study of Bjørklund et al. (2024), which included 228 individuals exposed to ionizing radiation as a result of activities at the Semipalatinsk Nuclear Test Site (SNTS), representing three generations from affected families [61]. The authors investigated polymorphisms in six genes associated with cardiovascular disease susceptibility. Notably, third-generation descendants demonstrated a higher prevalence of homozygous variants of the eNOS T786C and PON1 Gln192Arg polymorphisms (46.1% and 66.7%, respectively). These genetic variants have previously been associated with endothelial dysfunction, increased vascular tone, elevated blood pressure, atherosclerosis, dyslipidemia, and insulin resistance. Although causal relationships cannot be established, these findings suggest that genetic and epigenetic mechanisms may contribute to the long-term cardiovascular consequences observed in radiation-exposed populations. Similar biological pathways may also be relevant to the increased prevalence of cardiovascular diseases observed in the present study.

In our study, the most pronounced associations were observed among individuals exposed at younger ages, including those who received relatively low radiation doses. This observation may reflect increased susceptibility to radiation during critical periods of growth and development and supports the need for age-stratified analyses in future investigations. At the same time, it is important to recognize that the exposure conditions experienced by populations residing near the SNTS differed substantially from those reported in other radiation-exposed cohorts. Residents of affected territories were subjected to prolonged combined external and internal exposure over several decades, often beginning in early childhood. Such exposure patterns may have resulted in unique long-term health effects that are not directly comparable to those observed following acute radiation events.

Of particular interest is the observed burden of chronic diseases among individuals born after the cessation of nuclear testing who were not directly exposed to radiation. Although the mechanisms underlying these findings remain unclear, they highlight the importance of continued investigation of potential long-term and intergenerational health effects in populations residing near the SNTS. These individuals currently constitute a substantial proportion of the region’s working-age and reproductive-age population and therefore represent an important target group for preventive healthcare strategies and long-term medical surveillance.

### Strengths and Limitations

A major strength of the present study is the use of data from the State Scientific Automated Medical Registry (SSAMR), which contains detailed demographic, genealogical, medical, and dosimetric information for exposed individuals and their descendants. The availability of family linkage data across multiple generations and individual dose estimates substantially enhances the value of the analyses. However, several limitations should also be acknowledged. First, the cross-sectional design precludes causal inference and does not allow calculation of person-years at risk or direct estimation of disease incidence. Furthermore, the design of the present cross-sectional study does not allow direct examination of the mechanisms of intergenerational or transgenerational transmission of radiation exposure effects. Second, despite the availability of individual dose estimates, residual confounding by unmeasured environmental, lifestyle, or socioeconomic factors cannot be excluded. The high percentage of study participants’ refusal to answer the question about alcohol consumption is due to the peculiarities of local mentality, traditions and religious beliefs (the majority of study participants were Muslims). The disease prevalence comparisons presented in Table 4 are based on unadjusted analyses and may therefore reflect differences in demographic and other unmeasured characteristics between the study groups. Although the subsequent multivariable regression models were adjusted for age, sex, and smoking status, the possibility of residual confounding, including that related to socioeconomic factors, cannot be completely excluded.

Finally, the findings related to thyroid function were not entirely consistent across analytical approaches. Although the thyroid hormone measurements were verified against the original laboratory records and a sensitivity analysis excluding statistical outliers did not reveal any substantial influence on the results, repeat biochemical measurements were not available. Therefore, the observed differences in FT3 and FT4 levels should be interpreted with caution. Therefore, further studies incorporating thyroid ultrasonography, advanced endocrine assessments, and, where clinically indicated, histopathological evaluation are warranted to clarify the long-term effects of radiation exposure on thyroid health.

## 5. Conclusions

More than 35 years after the cessation of nuclear testing, the findings of this screening study suggest a persistent excess burden of major chronic diseases among populations residing in radiation-affected territories of Kazakhstan. Individuals from exposed areas demonstrated a higher prevalence of arterial hypertension, chronic ischemic heart disease, valvular heart disease, ischemic stroke, chronic cerebrovascular disease, thyroid disorders, malignant neoplasms, and gastrointestinal diseases.

Multivariable regression analyses further demonstrated dose-dependent associations between radiation exposure and coronary heart disease and thyroid disorders. Receiver operating characteristic (ROC) analysis indicated that radiation dose had good discriminatory performance for several of these outcomes, particularly cardiovascular diseases. Although the cross-sectional nature of the study precludes causal inference, the observed dose–response relationships support the hypothesis that long-term radiation exposure may contribute to the development of chronic non-communicable diseases in populations living near the Semipalatinsk Nuclear Test Site.

These findings highlight the importance of continued epidemiological surveillance, targeted screening programs, and long-term medical follow-up of both exposed individuals and their descendants.

## Figures and Tables

**Table 1:** Radiation doses among people included to the study group.

Birth Cohort	N	Median Dose, mSv (IQR)	Min; Max, mSv
Born before 1962	411	454.0 (257.0–789.0)	0; 1000
Born between 1963 and 1989	795	118.0 (56.0–158.0)	0; 676.0
Born after 1990	152	0.0 (0.0–0.0)	0; 7.14

**Table 2:** Reference (normative) values of biochemical blood parameters determined on the EXC 200 analyser (PRC, 2023).

Rate	Units (EXC 200)	Reference Values
GLU	mmol/L	3.90–6.10
UREA	mmol/L	1.70–8.30
CREA	μmol/L	44–97 (male)/35–80 (female)
TBIL	μmol/L	3.40–20.50
DBIL	μmol/L	<6.89
ALP	U/L	Male: 45–125; Female: age 20–49: 35–100; age 50–79: 50–135
ALT	U/L	Male: 9–50; Female: 7–40
AST	U/L	<40
GGT	U/L	Male: 11–50; Female: 7–35
CHOL	mmol/L	<5.20
TG	mmol/L	<2.30
UA	μmol/L	Female: 140–380/Male: 202–416

**Table 3:** Socio-demographic characteristics of the study participants.

Parameter	Study Group N (%)	Control Group N (%)
Age	54.0 (40.0–63.0)	53.0 (41.0–62.0)
Gender: female	1005 (74.0)	1234 (85.4)
Education: secondary	505 (37.2)	573 (39.7)
secondary specialized	664 (48.9)	491 (34.0)
higher	189 (13.9)	380 (26.3)
There are no occupational hazards	1269 (93.4)	1270 (91.0)
Marital status: married	1153 (84.9)	1043 (72.2)
Smoking: Never smoked	1130 (83.2)	1205 (83.4)
Smokes	72 (5.3)	141 (9.8)
Quitted smoking over a year ago	156 (11.5)	98 (6.8)
Alcohol: never consumed	589 (43.4)	765 (53.0)
currently consumed	91 (6.7)	197 (13.6)
consumed in the past	97 (7.1)	116 (8.0)
not specified	581 (42.8)	366 (25.3)

**Table 4:** Structure of diseases diagnosed during screening.

Disease	Study Group N (%)	Control Group N (%)	*p*
Hypertension	850 (62.6)	539 (37.3)	<0.001
Chronic ischemic heart disease	68 (5)	17 (1.2)	<0.001
Other heart diseases	79 (5.3)	43 (2.9)	0.001
Hemorrhagic stroke	7 (0.5)	5 (0.3)	0.774
Myocardial infarction	28 (1.9)	34 (2.3)	0.518
Heart valve disease	35 (2.3)	0 (0.0)	<0.001
Ischemic stroke	21 (1.4)	8 (0.5)	0.025
Chronic cerebrovascular diseases	21 (1.4)	5 (0.3)	0.003
Thyroid disorders	266 (19.6)	177 (12.3)	<0.001
Liver diseases	126 (8.4)	97 (6.5)	0.053
Diabetes mellitus	93 (6.2)	147 (9.8)	<0.001
Breast diseases	17 (1.1)	30 (2.0)	0.077
Malignant neoplasms	33 (2.2)	12 (0.8)	0.003
Gastrointestinal diseases	198 (13.2)	128 (8.5)	<0.001
Renal diseases	170 (11.3)	128 (8.5)	0.013
Respiratory diseases	79 (5.3)	30 (2.0)	<0.001

**Table 5:** Characteristics of biochemical parameters in the study groups.

Parameter	Study Group (Me; Q1–Q3)	Control Group (Me; Q1–Q3)	*p*
UA	199.9 (137.5–261.7)	239.6 (193.3–296.7)	<0.001
DBIL	2.1 (2.0–2.7)	2.1 (2.0–2.8)	0.078
TBIL	4.0 (3.1–5.8)	3.9 (3.1–5.2)	0.043
CREA	48.3 (35.6–59.7)	61.2 (53.8–71.1)	<0.001
UREA	4.5 (3.4 to 5.7)	5.3 (4.5–6.3)	<0.001
ALP	59.4 (38.7 to 81.0)	62.0 (44.1–82.3)	0.002
AST	17.6 (12.4 to 23.2)	21.9 (17.5–30.1)	<0.001
GGT	17.3 (11.4 to 28.3)	21.6 (14.7 to 34.3)	<0.001
GLU	4.6 (4.1 to 5.3)	4.9 (4.4 to 5.6)	<0.001
ALT	8.9 (5.5 to 14.4)	14.0 (9.4 to 20.3)	<0.001
CHOL	5.4 (4.7 to 6.0)	4.9 (4.0 to 5.8)	<0.001
TG	1.5 (1.1 to 2.0)	1.2 (0.9 to 1.9)	<0.001
TYG	8.2 (7.8 to 8.6)	8.1 (7.7 to 8.6)	0.003

Mann–Whitney U.

**Table 6:** Characteristics of the thyroid status.

Parameter	Study Group, Me (IQR)	Control Group, Me (IQR)	*p*
Anti-TPO	14.7 (5.0 to 43.2)	5.0 (5.0 to 12.9)	<0.001
TSH	1.5 (0.8 to 3.1)	0.8 (0.5 to 1.5)	<0.001
FT3	2.8 (0.8 to 4.8)	2.6 (2.1 to 4.1)	<0.001
FT4	9.6 (1.3 to 14.2)	10.6 (8.3 to 12.6)	<0.001

Anti-TPO—Anti-thyroid peroxidase autoantibodies; TSH—Thyroid-stimulating hormone; FT3—Free Triiodothyronine; FT4—Free Thyroxine.

**Table 7:** Laboratory parameters depending on age in the study group.

Parameter	Study Group, by Date of Birth (Me (Q1–Q3))			*p* *
	Before 1962 (N = 411)	1963–1989 (N = 795)	After 1990 (N = 152)	
GLU	4.67 (4.1–5.4)	4.63 (4.065–5.31)	4.67 (4.118–5.31)	0.01 * *p*1-2 = 1.0 * *p*1-3 = 0.152 * *p*2-3 = 0.436
CREA	49.10 (35.05–59.3)	48.20 (36.3–59.1)	56.05 (41.3–65.22)	0.167
CHOL	5.56 (4.995–6.13)	5.32 (4.54–5.95)	5.30 (4.415–6.54)	0.95
TG	1.67 (1.235–2.08)	1.41 (0.992–1.9)	1.38 (0.975–2.04)	0.198
TYG	8.32 (7.998–8.67)	8.15 (7.766–8.48)	8.25 (7.702–8.6)	0.547
Anti-TPO	7.836 (5.0–34.56)	15.649 (5.0–44.66)	15.78 (5.0–24.12)	0.106
TSH	1.465 (0.871–3.03)	1.482 (0.838–3.06)	1.431 (0.732–3.63)	0.159
FT3	2.442 (0.340–4.44)	2.849 (1.22–4.84)	3.729 (2.227–4.95)	<0.001 * *p*1-2 < 0.001 * *p*1-3 < 0.001 * *p*2-3 = 0.270
FT4	3.608 (0.966–13.16)	10.265 (1.482–14.3)	12.18 (9.610–15.17)	<0.001 * *p*1-2 < 0.001 * *p*1-3 < 0.0001 * *p*2-3 = 1.0

*p*—Kruskal–Wallis test. * Pairwise comparisons were performed using Dunn’s post hoc test with Bonferroni correction for multiple testing.

**Table 8:** Regression analysis of the relationship between radiation dose (OR per 10 mSv increase) and diseases.

Diseases	Crude OR; 95% CI	*p*	Adjusted OR *; 95% CI	*p*
Hypertension	1.104; 1.091–1.116	<0.001	1.01; 0.99–1.03	0.372
Chronic ischemic heart disease	1.026; 1.020–1.033	<0.001	1.0114; 0.99–1.0239	0.071
Myocardial infarction	1.009; 1.000–1.018	0.062	1.01; 0.99–1.038	0.062
Hemorrhagic strokes	1.022; 1.007–1.038	0.005	1.011; 0.991–1.0321	0.288
Ischemic strokes	1.026; 1.017–1.037	<0.001	1.007; 0.976–1.0382	0.671
Thyroid disorders	1.007; 1.003–1.011	0.001	1.017; 1.009–1.025	<0.001
Malignant neoplasms	1.025; 1.016–1.033	<0.001	1.0; 0.987–1.01	0.737
Renal diseases	1.012; 1.007–1.016	<0.001	0.99; 0.98–1.00	0.652
Respiratory diseases	1.017; 1.010–1.023	<0.001	1.00; 0.99–1.01	0.33

* Correction for age, gender, and smoking status. Radiation dose was analyzed as a continuous variable and expressed per 1 cSv (10 mSv) increase. All models were adjusted for age, sex, and smoking status. For binary outcomes, odds ratios (ORs) with 95% confidence intervals (95% CIs) are presented. The reference category was the absence of the corresponding disease.

**Table 9:** AUC in the model of disease development in exposed people.

Outcome	AUC	Std. Error	95% Confidence Interval		*p*
			Lower	Upper	
Hypertension	0.715	0.00879	0.698	0.732	<0.001
Chronic ischemic heart disease	0.735	0.0305	0.675	0.795	<0.001
Myocardial infarction	0.512	0.0378	0.438	0.586	0.744
Hemorrhagic strokes	0.643	0.0922	0.462	0.823	0.122
Ischemic strokes	0.711	0.0548	0.604	0.819	<0.001
Chronic cerebrovascular diseases	0.700	0.0515	0.599	0.801	<0.001
Thyroid disorders	0.540	0.0138	0.513	0.567	0.004
Breast diseases	0.468	0.0370	0.395	0.540	0.381
Gastrointestinal diseases	0.596	0.0167	0.563	0.628	<0.001
Renal diseases	0.574	0.0171	0.540	0.607	<0.001
Respiratory diseases	0.646	0.0273	0.592	0.700	<0.001
Malignant neoplasms	0.673	0.0442	0.586	0.760	<0.001

**Table 10:** Logistic regression analysis of the relationship between radiation dose and diseases.

Outcome	Dose, (N)	Adjusted OR (95% CI) *	*p*-Value
Hypertension	0 (reference), (572)	1.0	-
	<500 mSv, (634)	3.61 (2.96–4.39)	<0.001
	500–1000 mSv, (183)	9.88 (3.95–24.68)	<0.001
Chronic ischemic heart disease	0 (reference), (22)	1.0	-
	<500 mSv, (39)	2.66 (1.548–4.593)	<0.001
	500–1000 mSv, (24)	3.44 (1.737–6.81)	<0.001
Myocardial infarction	0 (reference), (39)	1.0	-
	<500 mSv, (14)	0.56 (0.304–1.05)	0.076
	500–1000 mSv, (9)	1.66 (0.70–3.95)	0.245
Hemorrhagic stroke	0 (reference), (5)	1.0	-
	<500 mSv, (4)	1.309 (0.34–5.01)	0.694
	500–1000 mSv, (3)	6.68 (0.98–45.2)	0.051
Ischemic strokes	0 (reference), (9)	1.0	-
	<500 mSv, (10)	1.84 (0.73–4.58)	0.193
	500–1000 mSv, (10)	6.15 (1.99–18.91)	0.002
Chronic cerebrovascular diseases	0 (reference), (7)	1.0	-
	<500 mSv, (17)	6.26 (2.35–16.63)	<0.001
	500–1000 mSv, (2)	6.79 (0.99–46.59)	0.051
Thyroid disorders	0 (reference), (224)	1.0	-
	<500 mSv, (175)	1.421 (1.138–1.775)	0.002
	500–1000 mSv, (44)	2.747 (1.798–4.196)	<0.001
Breast diseases	0 (reference), (30)	1.0	-
	<500 mSv, (15)	0.97 (0.514–1.85)	0.941
	500–1000 mSv, (2)	0.62 (0.134–2.87)	0.542
Malignant neoplasms	0 (reference), (15)	1.0	-
	<500 mSv, (16)	1.72 (0.839–3.544)	0.138
	500–1000 mSv, (13)	3.52 (1.45–8.54)	0.005
Gastrointestinal diseases	0 (reference), (145)	1.0	-
	<500 mSv, (142)	1.71 (1.32–2.2)	<0.001
	500–1000 mSv, (32)	1.65 (1.03–2.64)	0.037
Renal diseases	0 (reference), (142)	1.0	-
	<500 mSv, (119)	1.41 (1.08–1.83)	0.009
	500–1000 mSv, (35)	2.09 (1.31–3.32)	0.002
Respiratory diseases	0 (reference), (35)	1.0	-
	<500 mSv, (53)	2.22 (1.42–3.45)	<0.001
	500–1000 mSv, (17)	2.46 (1.22–4.98)	0.012

OR is the odds ratio; CI is the confidence interval. Logistic regression models were constructed separately for each outcome. The reference category for radiation dose was 0 mSv. N—number of participants with the disease. * Models were adjusted for age, sex and smoking status. N of reference group = 165: N of the group with radiation dose <500 mSv = 1.005; N of the group with radiation dose 500–1000 mSv = 188.

## Data Availability

The raw data supporting the conclusions of this article will be made available by the authors on request. The data are not publicly available due to privacy restriction.
